# Online Calibration of Extrinsic Parameters for Solid-State LIDAR Systems

**DOI:** 10.3390/s24072155

**Published:** 2024-03-27

**Authors:** Mark O. Mints, Roman Abayev, Nick Theisen, Dietrich Paulus, Anselm von Gladiss

**Affiliations:** Active Vision Group, Institute for Computational Visualistics, University of Koblenz, 56016 Koblenz, Germany

**Keywords:** solid-state LIDAR, extrinsic calibration, sensor fusion

## Abstract

This work addresses the challenge of calibrating multiple *solid-state* LIDAR systems. The study focuses on three different *solid-state* LIDAR sensors that implement different hardware designs, leading to distinct scanning patterns for each system. Consequently, detecting corresponding points between the point clouds generated by these LIDAR systems—as required for calibration—is a complex task. To overcome this challenge, this paper proposes a method that involves several steps. First, the measurement data are preprocessed to enhance its quality. Next, features are extracted from the acquired point clouds using the *Fast Point Feature Histogram* method, which categorizes important characteristics of the data. Finally, the extrinsic parameters are computed using the *Fast Global Registration* technique. The best set of parameters for the pipeline and the calibration success are evaluated using the normalized root mean square error. In a static real-world indoor scenario, a minimum root mean square error of 7 cm was achieved. Importantly, the paper demonstrates that the presented approach is suitable for online use, indicating its potential for real-time applications. By effectively calibrating the *solid-state* LIDAR systems and establishing point correspondences, this research contributes to the advancement of multi-LIDAR fusion and facilitates accurate perception and mapping in various fields such as autonomous driving, robotics, and environmental monitoring.

## 1. Introduction

Currently, spinning 360° LIDAR systems, referred as *classic* LIDAR systems in the literature, are established sensors for measuring distances and mapping, primarily in mobile systems applications such as vehicles or robots. Data are recorded by a full rotation of the sensor or an internal component such as a prism. The novel technology of *solid-state* LIDAR (SSL) dispenses with moving components to the greatest possible extent. Such sensors are robust against external influences such as vibration and shock [1]. In contrast to a 360° LIDAR, the field of view (FOV) of a fixed mounted SSL is limited to certain angles in vertical and horizontal direction, similar to a depth camera. Recorded measurements are passed to further processing as *point clouds*.

**Figure 1:**
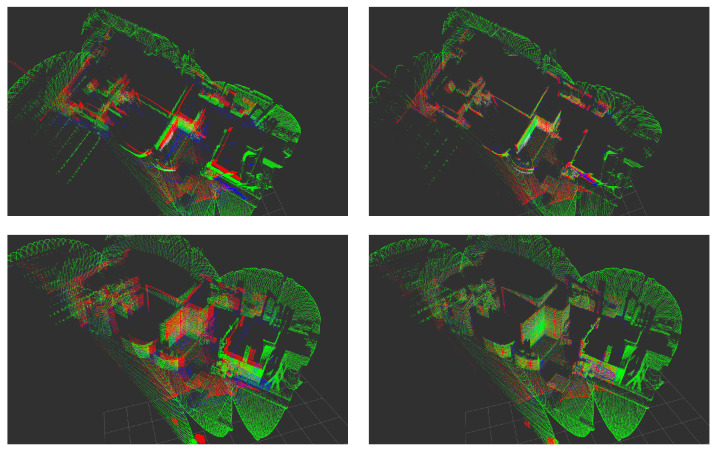
Example registration of point clouds provided by three SSLs: *Blickfeld Cube 1* (*red*), *Cepton Vista P-60* (*blue*), and *Livox Mid-100* (*green*). Shown is a scene that was recorded in a warehouse measuring 25 m×18.5 m. The upper images are in top-down perspective, the lower ones with a view from the front. On the left hand side, the initial point clouds of the individual sensors are shown. On the right, one finds the point clouds after successful calibration and transformation. The *Livox Mid-100* has been used as a target sensor and the point clouds of the other sensors have been registered to it.

The technologies that implement data acquisition of SSL sensors differ fundamentally. As a result, point clouds acquired by differently constructed SSLs correspondingly have manufacturer-specific characteristic properties and sampling patterns, see Section 3. Thus, the recorded point clouds cannot be registered to each other without additional effort. In this work, an online calibration pipeline is developed that is independent of interfering characteristics of the respective SSL systems, especially their distinct sampling patterns. First, Point Feature Histogram (PFH) [2,3] are computed to extract global features of point clouds based on local neighborhoods of individual data points. Then, the Fast Global Registration (FGR) [4] algorithm registers heterogeneous point clouds based on the PFH. This pipeline allows for extrinsic calibration of different sensors, and thus, enables further processing and application of fused sensor data. Specifically, a thorough evaluation of the different sensor systems regarding accuracy and resolution can be carried out in future work. Figure 1 shows the result of an exemplary calibration of three SSL sensors. The scene was captured in a warehouse measuring 25 m×18.5 m.

This work is structured as follows. Section 2 addresses related work dealing with extrinsic calibration of LIDAR systems. In addition to approaches based on SSL sensors, other related works mainly based on classical LIDARs were also examined. To gain insight into the utilized SSL systems, Section 3 presents the fundamental technologies of the sensors *Blickfeld Cube 1*, *Cepton Vista P-60*, and *Livox Mid-100*. After providing a background overview, the implemented calibration pipeline is shown in Section 4. Section 5 presents how the calibration system is evaluated. Finally, Section 6 summarizes the present work, followed by a final conclusion.

## 2. Related Work

The problem of extrinsic calibration of LIDARs can be fundamentally divided into two categories. On the one hand, there are systems relying only on LIDAR sensors and, on the other hand, mixed forms in which other sensor types, such as *RGB*-cameras, are fused with a LIDAR. These can be further divided into methods that rely on calibration patterns or those that do not. In the following, work is presented that has dealt with this problem and shows different approaches to solve it.

### 2.1. Classic LIDAR Systems

Zhou et al. [5] presented an automatic calibration of a classical LIDAR and a camera using a checkerboard pattern. Using line and surface detection, correspondences are created. In [6], calibration of two 360° LIDARs is performed using several commercial roadside traffic cones pasted with reflective film. Using the Random Sampling Consensus (RANSAC) algorithm [7], the reflective fringes of the cones in the point clouds of the sensors are extracted. Peters et al. in [8], performed extrinsic calibration for a lidar sensor and a robotic end effector the sensor is mounted on. By measuring and registering scans while the end effector is moving in a strictly defined way, it is possible to estimate the relative pose without any calibration pattern. For scan registration, the authors rely on the Iterative Closest Points (ICP) algorithm [9]. Jiao et al. [10] implemented an automatic calibration of multiple classical LIDARs on a vehicle in an urban environment. The goal was to perform the calibration independently of any external sensors, calibration patterns, and without prior knowledge regarding the environment. The authors formulated an optimization problem that was first tackled by an initial manual alignment of the sensors. In a second step, minima were found formulating a cost function based on a point-to-surface correspondence. These correspondences are determined utilizing the RANSAC algorithm. The final extrinsic parameters are determined via ICP.

### 2.2. Solid-State LIDAR Systems

Cui et al. [11] presented an automatic calibration procedure of a SSL (Livox Mid-40) to an RGB camera. However, this is based on a pattern recognition process, initialized by an image analysis of RGB data provided by the camera. Wei et al. [12] created a link between a SSL and a 2D 360° LIDAR. The goal was to perform Simultaneous Localization and Mapping (SLAM) on a mobile robot using both sensors. The calibration approach followed is based on the work by Kim and Park [6] and Jiao et al. [10]. A horizontally mounted reflective tape was used as reference pattern to detect the correspondence between the SSL and again a 2D 360° LIDAR. In an initial calibration, the robot was manually aligned so that the tape and the emission strip of the 2D 360° LIDAR were on top of each other. Then, an ICP registration was performed to merge the tape recorded by the SSL sensor and the measurement strip of the 2D 360° LIDAR. In the work of Liu and Zhang [13], a mobile robot equipped with nine SSL sensors, eight *Livox Mid-40s*, and one *Livox Mid-100* is presented. This way, a full 360° coverage by SSLs could be established. However, not all FOVs of the sensors overlap, e.g., one LIDAR is oriented to the front and one to the back of the robot. Therefore, a map-based method, presented by Lin et al. [14], is introduced. This approach combines the scans of all mounted SSLs to a fully accumulated point cloud, and hence, a 3D map. This map is then used as a registration base line for all LIDARs of the robotic system.

## 3. System Overview

This section shows how the data acquisition platform is designed in detail. The differences between the individual SSLs will be highlighted. The technology behind each sensor is explained, along with any unique features and limitations that each one has. An overview about some of the key characteristics of the sensors is given in Table 1. By the end of this section, the reader will have a thorough understanding of the hardware and technology used in this work. A comprehensive explanation and comparison of these technologies is given in [15].

### 3.1. Multi Sensor System

**Figure 2:**
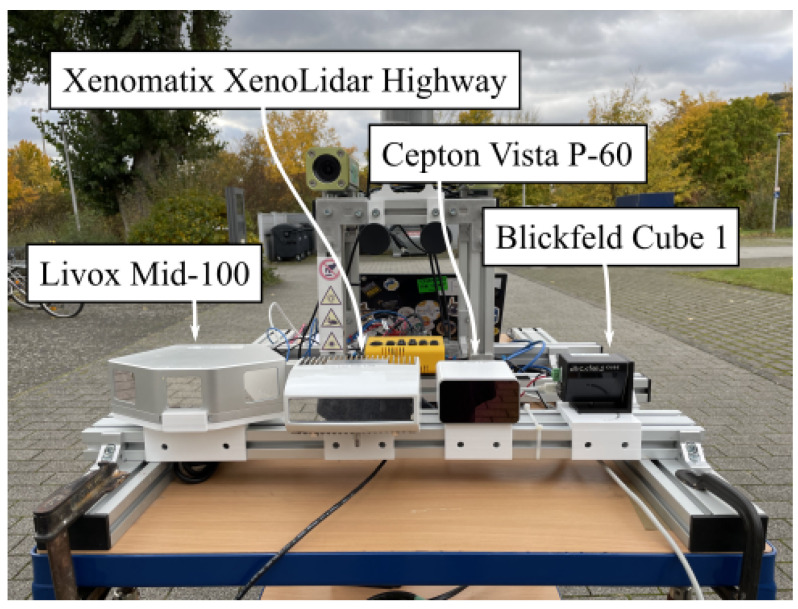
SSL sensors. The sensors are installed on a modular aluminum profile so that they can be quickly exchanged and expanded with additional sensors in the future. The setup is placed on a movable carriage. The sensor by Xenomatix is not used in this work due to restrictions regarding the acquisition parameters.

*Solid-state* LIDARs can be designed in different ways. A fundamental distinction must be made between *full-static* and *semi-static* implementations. Semi-static SSLs have moving components built in, despite the name. Full-static SSL systems are technical solutions that consist entirely of immovable components. Figure 2 shows the used sensors as they were mounted on the acquisition platform. The rack is designed so that additional sensors can be easily added. The sensors can be operated by battery or by main power, providing versatile options for different scenarios and ensuring continuous functionality. The setup is mounted on a carriage, so that the structure can also be moved manually. This flexibility has also allowed outdoor trips to be taken to record data and test the calibration approach presented in this work. In the following, the utilized SSL systems are shortly introduced from left to right. The *Livox Mid-100* is a LIDAR system that combines three *Livox Mid-40* sensors. On the second postion, a *Xenomatix XenoLidar Highway* is mounted. Due to the minimum measurement range (about 20 m) of this sensor and the comparably small FOV (30°×10°), it is not used in this work. However, the developed pipeline is fully compatible with the sensor. The third SSL in the series is the *Cepton Vista P-60*. A *Blickfeld Cube 1* finalizes the scanner array. All SSLs differ significantly in their technological hardware implementation. These differences on hardware level result in different dimensions of the FOV, different scanning distances and both heterogeneous and different sampling of the FOV. The following sections present each sensor in detail.

### 3.2. Livox Mid-100

**Figure 3:**
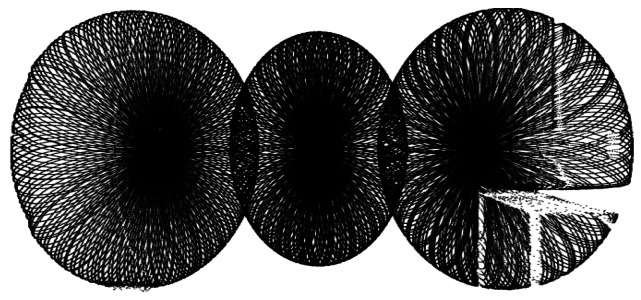
Scanning pattern of *Livox Mid-100*. The pattern is partially broken through occlusion (desk in the lower right corner).

The *Livox Mid-100* is a combined system consisting internally of three *Livox Mid-40* sensors. According to detailed study by Brazeal et al. [16], a Mid-40 sensor is based on the technology of *Risley prisms*. Such a semi-static SSL consists of two cylindrical prisms rotating around their own axis [17,18]. Figure 3 shows the resulting sampling pattern. It should be noted that these patterns are not repetitive. As a result, the resolution of the measurement increases with longer acquisition time. As the name implies, the horizontal opening angle of the Mid-100 is approximately 100°. This span is created by partially superimposing the installed *Mid-40s*, each with a 40° aperture angle. The circular scanning of the internal sensors results in an FOV of $98.4^{\circ }\times 38.4^{\circ }$. The range is specified as 130 m.

### 3.3. Cepton Vista P-60

**Figure 4:**
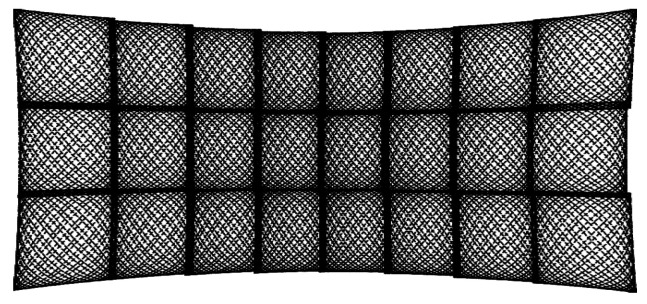
Scanning pattern of *Cepton Vista P-60*.

The basis of the semi-static SSL *Cepton Vista P-60* is the Micro Motion Technology (MMT) patented by the manufacturer. MMT is a novel development in the field of LIDARs. Optical components are set into fine micro oscillation to guide the laser beam through the FOV. The generated micro vibrations produce a mostly homogeneous scanning pattern. Figure 4 shows 3×8 patches, each with a pattern reminiscent of Lissajous figures. According to the manufacturer, the *Vista P-60’s* aperture angles are $60^{\circ }\times 22^{\circ }$ with a maximum viewing distance of 200 m.

### 3.4. Blickfeld Cube 1

**Figure 5:**
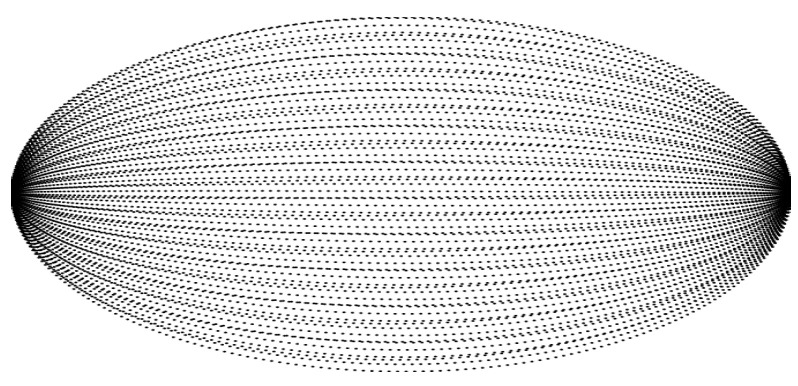
Idealized scanning pattern of *Blickfeld Cube 1*.

According to the manufacturer, the *Blickfeld Cube 1* is based on proprietary silicon mirrors of Micro Electromechanical Systems (MEMS). Movements are performed with very high precision [19]. Therefore, this sensor can be classified as a semi-static SSL. Two micrometer sized mirrors are vibrated to deflect a laser beam horizontally and vertically, spanning an FOV of $80^{\circ }\times 30^{\circ }$. Figure 5 shows the characteristic scanning pattern. By means of the installed technology, a range of 100 m can be achieved.

## 4. Methodology

To perform the implementation of our approach, the following algorithms and methods were used. The Fast Point Feature Histogram (FPFH) by Rusu et al. [2] and Rusu et al. [3] plays a central role. This method extracts global features of a point cloud based on local neighborhoods of individual data points. These properties are used for FGR [4], which is able to register heterogeneous point clouds based on feature extraction. The implemented software was deployed to the Robot Operating System (ROS) (https://www.ros.org/, accessed on 20 March 2024).

In the following, the background of the algorithms used in this work will be disclosed, which are decisive for the implementation of the extrinsic calibration. First, Section 4.1 specifies the objectives of our work. Then, Section 4.2 gives a brief insight into the preprocessing stage, before getting into the core algorithms. The following Section 4.3 discusses how features can be extracted from the sensor data. Section 4.4 describes how these features are processed to register the point clouds of the respective SSLs. The overall implementation of a calibration pipeline including an integration into ROS is presented in Section 4.5.

### 4.1. Calibration by Registration

In this work, point clouds from different SSL systems are calibrated by determining one target sensor and registering the data from the source or moving sensors to the data of the target sensor. Thus, the point clouds of the source sensors are calibrated to the coordinate system of the target sensor. In other words, the target sensor’s coordinate system spans the global coordinate system.

### 4.2. Preprocessing

**Figure 6:**
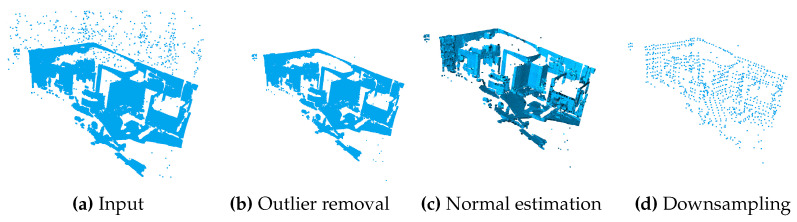
Input point cloud (**a**) and preprocessing steps 1 to 3 of the described calibration pipeline shown in (**b**–**d**).

Based on [20], the incoming data are first handled by a preprocessing stage. Figure 6 shows the individual steps. Since the point clouds delivered by the sensors are partially affected by noise and this has a clear effect especially at the outer rim of the point clouds, the outliers have to be removed in the first step. For this purpose, a statistical method is used. Then, the normal vectors for each point are determined from the neighborhoods of the other points. These are necessary for the feature extraction which is described in Section 4.3. The preprocessing is finished with a voxel-based downsampling. These voxels are used to average multiple points to one point. This reduces the density of the point cloud without disturbing the geometric structure.

### 4.3. Fast Point Feature Histogram

**Figure 7:**
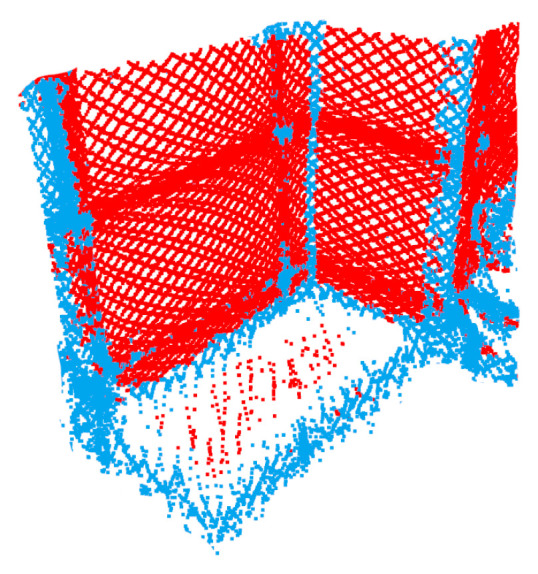
Example segmentation: point cloud of a room corner into edges (blue) and planes (red) using the FPFH algorithm. The point cloud has been recorded using a Cepton Vista-P60.

The PFH is a method for segmenting point clouds depending on the surface shapes on which the respective points were located at the time of acquisition [2]. These surface shapes are divided into seven categories: *cones, edges, spheres, planes, tori, corners*, and *cylinders*. To determine the geometric relation between the points of a point cloud, local features between each point and its *n* nearest neighbors are examined. Crucial information of such a cluster around a point are given by 3D coordinates and the normal direction of the neighboring points. Rusu et al. [3] have published a further development of the method called FPFH, which was utilized in our work. Comparing neighborhoods within a point cloud is a non-trivial problem and results in long runtimes. Through the optimized implementation, the authors were able to improve their earlier approach of PFH compared to FPFH from a runtime complexity based on *k* points from $O(k^{2})$ to O(k). Figure 7 shows an example of a segmented point cloud.

### 4.4. Fast Global Registration

Zhou et al. [4] presented in their work an algorithm for global registration of partially overlapping point clouds called FGR. This method promises more accurate registration with more robustness to noise and less computation time than conventional algorithms.

To establish an initial correspondence of a point cloud P to a target point cloud Q, the features $F\left(P\right)=\left\{F(p):p\in P\right\}$ and F(Q)$=\left\{F(q):q\in Q\right\}$ of the clouds are determined by FPFH. For each point p∈P, nearest neighbors in F(q) are searched via the features F(p), equivalently in the direction from Q to P. This initial correspondence is now used to compute a rigid transformation T from P to Q. A key point here is that the authors have implemented a solution to the point cloud distance minimization optimization problem that is robust against noise.

For this purpose, the Euclidean distance ∥p−Tq∥ for all p∈P and q∈Q is computed. It should be noted that p and q must each be extended by a homogeneous component with the value 1 and be normalized in order to calculate a valid Euclidean distance. The calculation of the distance between all points of P to all points of Q is challenging. A common solution to this problem is the use of so-called *k*-d-trees. A *k*-d tree is a binary tree data structure that organizes data points and allows for rapid indexing. The name stands for *k* dimensions, i.e., for the *k* values per point of a set that is to be structured. In the case of the point cloud to be processed, *k* is, therefore, obviously equal to 3 for the coordinates, i.e., three dimensions, along the Cartesian coordinates *x*, *y*, and *z*, respectively. To calculate the distances between all p∈P to all points of a point cloud q∈Q, a nearest neighbor search algorithm has to be introduced. For this purpose, Zhou et al. [4] integrated Fast Library for Approximate Nearest Neighbors (FLANN) by Muja and Lowa [21], an optimized, highly parallelized solver for nearest neighbors search. To determine the feature matching, the calculated distances are then adjusted by the *Geman–McClure* [22] estimation function, an *M-estimator* [23] implementation. The function allows for smoothing jumps in distances caused by noisy data and maximizing true values.

### 4.5. Calibration Pipeline

The calibration is essentially based on a pairwise registration of two point clouds, which will be referred to as source cloud P and target cloud Q in the following. The pairs can be selected independently from the SSL sensor data. Both point clouds must undergo a series of preprocessing steps until the final extrinsic parameters are computed, further referred to as the *calibration pipeline*. Figure 6 visualizes the steps that are taken between points 1 and 3.

1. **Statistical Outlier Removal:** Noise from the sensors and possibly other external influences create outlier points that hinder further computation. These points must be filtered out of the input point cloud.
2. **Estimation of Point Normals:** To calculate the FPFH, normals must be determined for all points in P and Q by analyzing neighborhood points for each point [20].
3. **Downsampling:** The FOV is divided into a grid of equally sized cubic voxels. Points within the same voxel are combined into one representative point by averaging. For this, a method from the *Open3D* framework [20] is used. This aggregation step significantly reduces the number of points that need to be processed while preserving the overall spatial distribution and characteristics of the original data. Note that the origin of the coordinate system is maintained.
4. **Calculation of FPFH:** FPFHs containing the geometric properties of the clouds are computed for P and Q, respectively.
5. **Registration:** Now, the FGR is applied to P and Q. In addition to the extrinsic parameters, an error is also calculated, which can be used to evaluate the accuracy of the registration experiment.
6. **Returning the Extrinsic Parameters:** The last step is to output a transformation matrix $E_{PQ}\in R^{4\times 4}$ containing rotation and translation parameters, which are called extrinsic parameters. The transformation matrix $E_{PQ}$ is applied, i. e., multiplied, to each point p∈P to transform these into the target coordinate system of Q. A fourth homogeneous coordinate is priorly added to the three-dimensional points in P in order to perform the transformation.

**Figure 8:**
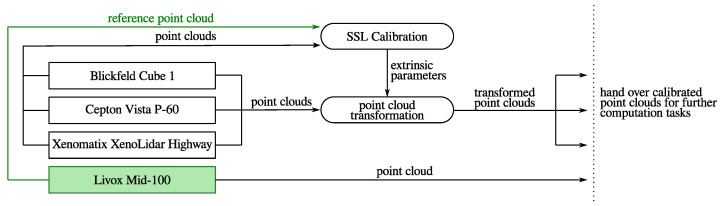
Visualization of the *ROS node* structure. The green marked sensor, here *Livox Mid-100*, symbolizes the target data for the calibration. The shapes with sharp corners represent nodes for data recording provided by *Solidar-Lib*. Shapes with soft corners denote nodes for data processing. Arrows indicate the data flow. Note that the sensor *Xenomatix XenoLidar Highway* has not been used in the experimental part of this work.

The data acquisition is performed by an internally developed software suite named *Solidar-Lib*, which is implemented in C++. The base of the calibration implementation is the *Open3D* framework. The focus is on the processing and manipulation of 3D sensor data, corresponding to point clouds. For integration into ROS, a *node* was implemented. Using this adapter node, sensor data are passed to the calibration pipeline and extrinsic parameters are returned as the return value. Data are passed between program components in the form of point clouds. The calibration runs on a notebook with a hexa-core processor, more precisely an *Intel i7-1255U* and 32 GiB RAM in the range of approximately one to three seconds. Another node can now transform the corresponding source cloud to the target cloud with these parameters. Figure 8 shows a schematic representation of the relations between the nodes involved. As an example, the *Livox Mid-100* has been marked as the target sensor. However, any other sensor of the system can be used as a target for calibration. Note that the listed *Xenomatix XenoLidar Highway* sensor has not been used in this work due to its acquisition parameters. However, it is fully compatible with the developed pipeline and ROS node.

## 5. Experimental Setup and Evaluation

This section deals with the evaluation of the presented system and the calibration pipeline. Details about the sensors are described in Section 3 and Table 1. We performed experiments at indoor conditions. In performing the experiments, we postulated that the intrinsic calibration of the respective sensors is correct.

First, Section 5.1 introduces a metric definition for evaluating the method. Second, an experiment to study the impact of voxel size to measurement error and runtime is shown in Section 5.2. The series of experiments is concluded with an experiment at outdoor conditions while moving the sensor system, which is described in Section 5.3.

### 5.1. Error Calculation and Metrics

**Figure 9:**
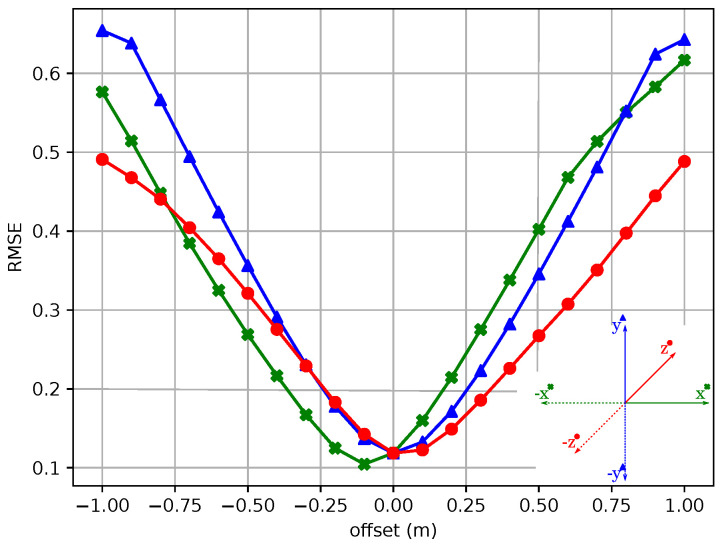
Plot of the RMSE as a function of the left and central sensor of the *Livox Mid-100*. The left sensor was synthetically shifted in x (green ‘x’), y (blue triangles), and z (red dots) direction, and hence, left-right, top-down, and front-back, respectively. The negative shift of the measurement along the x-axis is due to the fact that the left sensor has a larger overlap with the central target sensor when moving to the right.

The Root Mean Square Error (RMSE) is a parameter that specifies the ratio of an input dataset to a target dataset. In the case of calibration data analysis, the transformed source point cloud is compared to the target point cloud accordingly. According to Lehtola et al. [24], RMSE is a common method for comparing registration results. In the work on FGR [4], this error measure is also used to compare different algorithms. Lui and Zhang [13] used the default manufacturer’s calibration of the *Livox Mid-100* as ground truth to define a metric. The point cloud of one of the outer sensors was synthetically transformed. A correspondence value to the central SSL was then calculated using the error function. By knowing the exact displacement, a metric could, thus, be defined. In our work, a similar approach is taken. The left sensor of the *Livox Mid-100* is synthetically displaced in all three coordinate directions and subsequently compared with the center sensor. Figure 9 shows the variation of the displacement in relation to the RMSE. Without displacement, the error value is 0.115. With a shift of 25 cm the error is between 0.15 and 0.23, depending on the axis. Through this experiment, it is possible to establish a connection between a metric value and the comparative relation of RMSE.

### 5.2. Examination of Preprocessing Parameters

**Figure 10:**
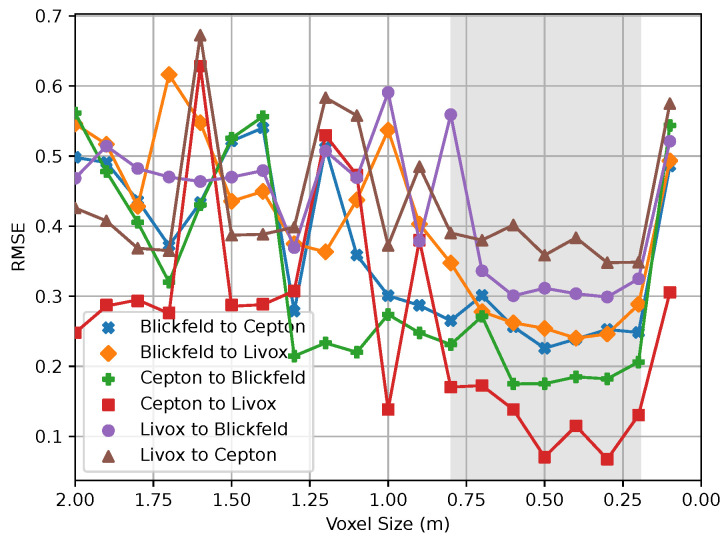
The impact of the voxel size (m) on the RMSE. It is evident that for all possible sensor pairings, the best results were measured in the range between 0.80 m and 0.20 m. The gray area highlights this region of interest in the plot.

**Figure 11:**
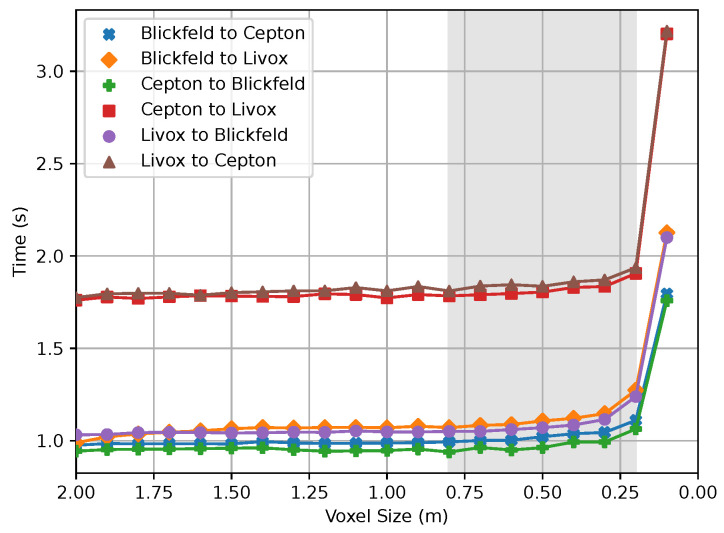
The impact of the voxel size (m) on the runtime (s). A voxel size of 0.2 m forms a boundary for all pairings before the runtime expands into a range no longer suitable for the intended use case. The gray area highlights this region of interest in the plot.

Considering the described metric and in addition the overall processing runtime, experiments were created with all possible combinations of origin and target point clouds. The results regrading the RMSE are presented in Figure 10. The time taken to perform the final transformation of the registration is shown in Figure 11. The calculation of the error function is performed on the source point clouds with removed outliers. When referring to voxel size, the value is used to mean the length of each edge of the cubic body. Among all combinations, there is a trend that smaller voxel sizes, starting from about 0.7 m, cause less error. However, there is also a tendency for deterioration from a voxel size of 0.2 m. It stands out that the order of a pairing plays a significant role. This discrepancy between the *Livox Mid-100* and *Cepton Vista P-60* sensors is particularly noticeable. When calibrating *Cepton* to *Livox*, the best overall result was obtained with an RMSE of 0.07. In contrast, the lowest RMSE, with a calibration from *Livox* to *Cepton* of 0.35, is five times as large.

Regarding the runtime, it can be interpreted that it does not show significant deviations in relation to the voxel size in the context of the individual experiments. However, there are differences regarding the runtime among the individual sensor combinations. In connection with the RMSE measurement, the range of voxel size between 0.7 m and 0.2 m deserves special attention. In contrast to the error measurement, there are no differences in the order of the respective pairings. However, a clear characteristic is the difference of the pair of sensors *Livox Mid-100* and *Cepton Vista P-60* to the pairings that include the *Blickfeld Cube 1*. There is a difference in the runtime of about 0.7 s. This difference can be explained by the fact that the original data of these sensors have more data points than the *Blickfeld*. Table 2 shows the number of points in the respective clouds in relation to the voxel size. Since the final transformation of the point clouds to perform the registration happens on the non-downsampled data, a longer computation time is needed for this final step.

Table 3 shows a summary of the best results in terms of error value and runtime for each of all possible sensor pairings. From the data, it can be concluded that using the *Livox Mid-100* as the central target sensor yields the most suitable results. In summary, it can be concluded that with a well-chosen voxel size and optimally chosen sensor pairing, the calibration runtime is less than four seconds in the specified evaluation environment. This runtime is sufficient to perform an extrinsic calibration during operation of the sensor system. Thus, the calibration can be rated as online-suitable.

### 5.3. Real-World Scenario

To assess the multi-sensor system for robustness against motion, the SSL system was relocated outside the local research facility. Subsequently, data were recorded while moving the multi-sensor system. After reviewing the recorded data, however, it had to be determined that a significant statement regarding robustness cannot be made. The main reasons for this are non-measurable, dynamic environmental components such as crossing people or vehicles. In addition, the structure was not moved constantly, or the movement was not measured in terms of pose, speed, and acceleration. Nevertheless, usable conclusions could be drawn from this experiment. To guarantee a stable extrinsic calibration of all SSL sensors, a synchronization of the respective sampling rates has to be introduced. Due to motion, the FOVs of the individual sensors change at different times. As a result, certain image areas do not correspond to the true pose of the sensors at the moment of data transmission to the calibration pipeline. This delay results in incorrect extrinsic parameters.

**Figure 12:**
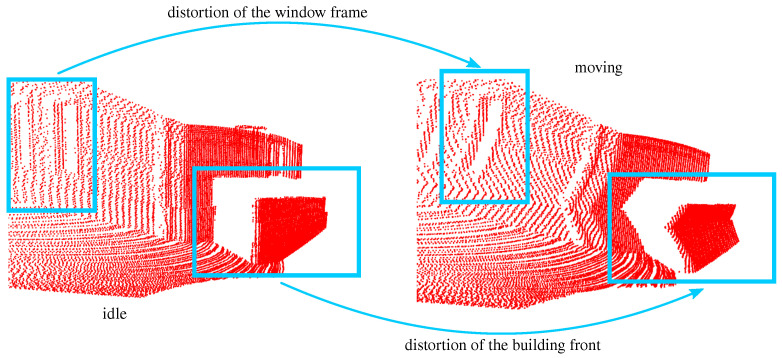
Representation of a distortion triggered by movement of the sensors during data acquisition. The data originate from the *Blickfeld Cube 1*.

In addition, motion leads to drifting effects, resulting in distorted point clouds. At low sampling rates and high motion speeds, this effect occurs severely. The *Blickfeld Cube 1* sensor is particularly affected. Figure 12 shows this effect in comparison to standstill. It can be clearly seen that the building front “bends away”. Another distortion can also be seen at window boxes in the left part of the figure.

A possible solution to this problem can be the incorporation of *odometry* [25]. This can be achieved via additional sensors, such as an Inertial Measurement Unit (IMU). Moreover, it is also possible to implement optical odometry via the existing SSL sensors. Rozenberszki and Majdik [26] presented such a method in their work. Using this odometric data, it is potentially possible to correct distorted point clouds.

## 6. Summary and Conclusions

In this research work, a method was presented with which it is possible to calibrate three different SSL sensors online extrinsically. The challenges of extrinsically calibrating different SSL sensors originates from the different technologies (MEMS, MMT, and Risley prisms) employed for data acquisition. Each SSL system has a characteristic sampling pattern due to these technologies. These technology-related patterns aggravate to perform a calibration because registration algorithms are heavily influenced by prominent features. A pipeline was introduced to process sensor data, which essentially performs the following steps: outlier removal, downsampling, feature extraction, and calculation of extrinsic parameters through registration. Through experiments that examined the implemented calibration system for accuracy, runtime, and robustness, it was shown that the used methods offer a potential solution. The calibration pipeline was implemented as a standalone package for ROS.

A new problem was uncovered by examining the system in motion. Moving the sensors while they acquire data can cause distortion of the resulting point clouds. Looking forward to future developments, this problem can be addressed using odometry.

In this work, the sensors have not been calibrated for their intrinsic parameters. It was assumed that the manufacturer’s intrinsic calibration would suffice. In future work, the intrinsic calibration of the sensors needs to be carried out, as this might enable more accurate results in fusing the point clouds.

## Figures and Tables

**Table 1:** Overview about a selection of key characteristics of the different SSL systems.

	Livox Mid-100	Cepton Vista P60	Blickfeld Cube 1
Technology	Risley prisms	MMT	MEMS
FOV	98° × 38°	60° × 22°	80° × 30°
Samples per second	300.000	315.000	270.000 *
Measurement range	1–260 m	5–200 m	5–250 m
Range precision ^†^	2 cm (@10 m)	3 cm (60%@6 m)	2 cm (50%@10 m)

* Depending on parameters of sampling pattern; ^†^ According to data sheets; reflectance and distance values are given in brackets.

**Table 2:** Number of points within the downsampled point clouds for each of the sensors *Blickfeld*, *Cepton*, and *Livox*. The voxel size entries of 0.0 m show the number of points in the clouds after the outlier removal process, but without any downsampling.

Voxel Size (m)	0.8	0.5	0.2	0.1	0.0
**Blickfeld**	463	1044	5613	16,068	32,926
**Cepton**	443	946	4267	13,958	190,971
**Livox**	684	1669	8273	26,946	261,041

**Table 3:** Optimally selected voxel size for all possible sensor combinations. The sensors were each abbreviated with the first letter of the manufacturer: B (*Blickfeld*), C (*Cepton*), and L (*Livox*). The fastest calibration was achieved with the C to B pairing. The most accurate calibration was achieved with C to L. The slowest and simultaneously the least accurate result was measured with the L to C experiment.

	B-C	B-L	C-B	C-L	L-B	L-C
**voxel size (m)**	0.5	0.4	0.6	0.3	0.3	0.3
**RMSE**	0.23	0.24	0.17	0.07	0.31	0.35
**time (s)**	1.02	1.12	0.95	1.84	1.11	1.87

## Data Availability

The data are not publicly available.

## Abbreviations

The following abbreviations are used in this manuscript:

SSL	*solid-state* LIDAR
FOV	Field of View
RANSAC	Random Sampling Consensus
ICP	Iterative Closest Points
SLAM	Simultaneous Localization and Mapping
MMT	Micro Motion Technology
MEMS	Micro Electromechanical Systems
FPFH	Fast Point Feature Histogram
FGR	Fast Global Registration
ROS	Robot Operating System
PFH	Point Feature Histogram
FLANN	Fast Library for Approximate Nearest Neighbors
RMSE	Root Mean Square Error
IMU	Inertial Measurement Unit
